# “Mens Sana in Cute Sana”—A State of the Art of Mutual Etiopathogenetic Influence and Relevant Pathophysiological Pathways between Skin and Mental Disorders: An Integrated Approach to Contemporary Psychopathological Scenarios

**DOI:** 10.3390/cells12141828

**Published:** 2023-07-12

**Authors:** Vincenzo Papa, Federica Li Pomi, Francesco Borgia, Sara Genovese, Giovanni Pioggia, Sebastiano Gangemi

**Affiliations:** 1 School and Operative Unit of Allergy and Clinical Immunology, Department of Clinical and Experimental Medicine, University of Messina, 98122 Messina, Italy; papavi.994@gmail.com (V.P.); sebastiano.gangemi@unime.it (S.G.); 2 Department of Clinical and Experimental Medicine, Section of Dermatology, University of Messina, 98122 Messina, Italy; federicalipomi@hotmail.it; 3 Institute for Biomedical Research and Innovation (IRIB), National Research Council of Italy (CNR), 98164 Messina, Italy; sara.genovese@cnr.it (S.G.); giovanni.pioggia@cnr.it (G.P.)

**Keywords:** mental health, mental illness, oxidative stress, inflammation, skin, psoriasis, atopic dermatitis

## Abstract

The negative socioeconomic impact of mental health disorders and skin diseases has increased in part due to the conflict between Russia and Ukraine, which has been a fertile ground for the emergence of psychopathologies. It is firmly established that there is a direct thread of etiopathogenetic communication between skin diseases and neuropsychiatric disorders, and the literature has tried to reveal the pathophysiological mechanisms governing such bidirectionality. This paper discusses this complex network of molecular pathways that are targeted by conventional and biological pharmacological agents that appear to impact two pathological spheres that previously seemed to have little connection. This molecular discussion is supplemented with a literature review, from a clinical viewpoint, regarding skin–brain etiopathogenetic bidirectionality. We focus on post-traumatic stress disorder (PTSD), which can be considered for all intents and purposes a systemic inflammatory disease that also affects the skin. A brief overview is also provided on the diagnostic–therapeutic and follow-up potential of oxidative and inflammatory markers potentially involved in the pathophysiological mechanisms treated. The aim is to clarify how these mechanisms may be useful in defining different stress-coping strategies and thus individual phenotypes of stress sensitivity/resistance in order to promote personalized medicine in the field of psychodermatology.

## 1. Introduction

### 1.1. Physical and Mental Illnesses: An Etiopathogenetic Bidirectionality on a Large Clinical and Anagraphic Scale

The Latin locution “Mens sana in corpore sano” of Juvenal lets us well understand that since the origins of civilization, the well-being of mind and body have been the highest aspiration of all mankind. Furthermore, in the most modern holistic reinterpretation of this locution, the importance of a close functional correlation between physical and mental health is recognized, enabling us to speak precisely of psychophysical unity. The mutual influence of the etiopathogenetic correlation between system-wide chronic diseases and several psychological conditions, such as anxiety, depression, psychological stress, and post-traumatic stress disorder (PTSD), has frequently been investigated in a clinical setting [1]. Although organic pathological states, with their social burden of related disabilities, are likely to occur in association with the onset of mental disorders, more intriguing and for that reason increasingly investigated is the triggering role of various psychological states in the onset of the most diverse organic diseases. The literature has no shortage of evidence concerning cardiovascular, metabolic, gastrointestinal, vestibular, neurodegenerative, autoimmune, and oncological diseases [2,3,4,5,6,7,8,9,10,11,12,13,14]. This reciprocal influence of mental health on physical health also exists in old age, and a high psychological resilience in the elderly appears to correlate with a reduced risk of developing age-related chronic diseases, thereby promoting physical resilience. How the low psychological resilience relates to chronic psychological stress exacerbates the impact of gradually increasing age-related chronic systemic inflammation (the so-called “inflammaging”) has also been investigated, especially given that, along with oxidative stress (OS) mechanisms, it can lead to the onset of age-related chronic conditions [15,16]. Similar evidence is also found in young people. In fact, during childhood, early exposure to severe chronic stressors has been associated with life-long negative consequences on both physical and mental health. These findings seem to point to the existence of a neuro-immune network based on a bidirectional crosstalk enhanced by early adverse events, in which inflammation would play a crucial role in bridging mental and physical disorders [17].

### 1.2. PNEI: A Symphonic Inter-Systemic Molecular Orchestra?

Such aspects of etiopathogenetic bidirectionality discussed until now find broader underpinnings in the holistic discipline of psycho-neuro-endocrine immunology (PNEI), which recognizes a non-negligible homology in receptors and molecular messengers and thus an intimate cross-influence between the psycho-neuro-endocrine system and immune response in a disease context [18]. Therefore, assuming an outdated dichotomy between biology and psychology, as also exemplified by the placebo effect [19], the most striking clinical implications PNEI have been found in the oncological field, particularly regarding perturbation in specific cellular signaling patterns [20] as well as the establishment of specific T lymphocyte alterations favoring tumor progression in cancer patients phenotyped for a specific psychological profile [21]. Preliminary assessments of psychological–spiritual states in such patients thus play an important prognostic role in terms of the effectiveness of immunochemotherapy treatment [22]. The scientific relevance of PNEI has been supported through clinical trials, both controlled and randomized, on the efficacy of short-term meditative practices based on it [23,24] as well as during COVID-19 pandemic [25] but also in non-clinical settings [26]. In this complex network of functional interactions subtending PNEI, to which melatonin appears to be a pleiotropically key chemical messenger [27], an increasingly recognized core anatomical role has been ascribed to the hypothalamocerebellar circuit [28]. The promising anti-inflammatory and antioxidant role of melatonin through the regulation of Forkhead box O3 (FOX03A) signaling at the astrocytic and microglial levels has also recently been highlighted. These pathways involve the melatonin transmembrane receptors MT1 and MT2, with interesting therapeutic implications in its possible use for neuroprotective purposes in major depressive disorder and in cognitive disorders. On the other hand, astrocytic and microglial activation and thus neuroinflammation mediated by type-17 signature cytokines appear to be triggered by the interaction of this hormone with some of its nuclear receptors, such as retinoid-related orphan receptor alpha and gamma (ROR-α and ROR-γ) [27]. Even more interesting, from a purely mechanistic perspective, are the most recent advances in recognizing that epigenetic regulation pathways are the key to directing the molecular translation from the psychic to the biological world [29].

Within the general topic of etiopathogenetic bidirectionality between systemic organic disorders and mental ailments, the skin deserves a separate mention due to its ectodermal embryological origin in common with the whole nervous system [30].

### 1.3. Skin as Stress-Triggered Neuroimmunoendocrine Organ

The direct communication between the skin and the neural system is key to the newly revised notion of cutaneous neurogenic inflammation (CNI), a pathophysiological phenomenon common to various chronic inflammatory skin diseases such as atopic dermatitis, psoriasis, rosacea, prurigo nodularis, hypertrophic scars, and sensitive skin [31]. CNI is based on a complex communication network between nerve endings and various skin cells, both immune and structural, through the direct involvement of neuropeptides, neurotrophins, proinflammatory cytokines, and vasoactive amines, which interact with specific receptors through feedback mechanisms leading to cutaneous neuroinflammation. In this neuroimmune crosstalk, a crucial signaling role is played by mast cells (MCs), which are known as “gatekeepers” because of their proximity to nerve endings. Mental stress impacts this network by activating the peripheral analog of the hypothalamic–pituitary-adrenal (HPA) axis in the skin [32], thus essentially making the skin a neuro-immuno-endocrine organ [31].

The skin represents both the target and the local source of pivotal stress mediators including the corticotropin-releasing hormone (CRH), the adrenocorticotropic hormone (ACTH) with its pre-prohormonal form proopiomelanocortin (POMC), and cortisol. This means that there is a peripheral CRH–POMC–ACTH–corticosteroids axis that is crucial in the skin response to psychological and emotional stress. Specifically, the pathway that is activated in the presence of such stressor stimuli involves the initial secretion of CRH by epidermal keratinocytes and dermal fibroblasts, which in an autocrine manner, upon interaction with specific CRH receptor type 1 (CRH-R1), induces the production of POMC and then the secretion of ACTH. After interaction with the melanocortin receptor (MCR), this leads to a downstream release of cortisol and corticosterone by the same cells in addition to the secretion of steroid hormones, including estrogens and androgens. From the complex interaction between these hormonal mediators and the skin’s cellular immune network as well as the mutual activating influence between the central HPA and the local skin, the CRH–POMC–ACTH–corticosteroids axis gives rise to inflammation and impairs the skin’s integrity and healing, thus inducing various stress-related skin diseases [33].

Indirect confirmation of this is the anti-inflammatory potential of antidepressants in the treatment of the most common inflammatory skin diseases associated with psychiatric comorbidities. From a pharmacodynamic perspective, the two endocrine-immunomodulatory mechanisms involved would act on preventing activation of the HPA axis (and presumably of its peripheral cutaneous analogue) as well as on containing the release of pro-inflammatory cytokines in favor of immune-regulatory ones [34]. There is thus a clear link between stress and/or the most common neuropsychiatric disorders and inflammatory skin diseases. In their review, McPhie et al. highlighted various inflammatory dermatoses and autoimmune skin diseases commonly comorbid with schizophrenia [35], and aspects of daily socio-economic life are being increasingly investigated in this light. For example, shift work disrupts the circadian system (which virtually controls all bodily functions), often leading to chronic stress and poor sleep. These in turn trigger the onset of chronic systemic inflammation and autoimmune, infectious, oncological, and allergic skin diseases via impaired innate and acquired immunity [36].

### 1.4. Skin Disease-Related Pro-Inflammatory Habitus as a Trigger for Mental Disorders

Given these premises and the cornerstones of psychodermatology regarding mutual etiopathogenetic influence between mental and skin disorders, on the other side of what has been so far pointed out, the triggering role of skin diseases’ pro-inflammatory habitus in the occurrence of mental disorders in such patients is also not negligible [37]. In fact, anxiety and depression are commonly found in patients with inflammatory skin diseases and of such clinical relevance that they cannot be justified by the mere psychosocial impact accompanying such disfiguring disorders. In this regard, the pathway involved likely causes pro-inflammatory cytokines to be produced at the skin level, primarily tumor necrosis factor-α (TNF-α) and interleukin-6 (IL-6). The latter, together with dendritic cells, cross a blood–brain barrier already weakened by stress with relative ease, due in particular to psychological stress-induced excitotoxicity (glutamatergic hypertone) followed by the upregulation of the proinflammatory cytokine signaling and subsequent generation of reactive oxygen species (ROS), resulting in oxidative damage [38,39]. Once reaching the central nervous system, these mediators would trigger a cascade of molecular signaling events. These events actively involve microglia in triggering the downregulation of synaptic and thus neurotransmitter function (with emphasis on the serotonergic metabolism) as well as reduced neurogenesis in several cerebral regions, including the hippocampus, which is implicated in the pathogenesis of depression [40]. Dysregulation of the kynurenine pathway is also part of the etiopathogenetic definition of the inflammatory theory of depression. Due to the inflammation-induced enzymatic hyperactivity of indoleamine-2,3-dioxygenase (IDO) at the microglial, neuronal, and astrocytic levels, tryptophan is catabolized to kynurenine, a neurotoxic metabolite responsible for neurodegenerative processes, at the expense of its utilization for the production of serotonin and melatonin, which are key hormones in mood regulation [41].

Key to the etiopathogenetic findings discussed so far is the dysfunctional role of a recently discovered network or, more properly, the supplementary circulation system: the glymphatic system. This system is a functional analogue of the peripheral lymphatic system. It is essential in maintaining cerebral homeostasis by draining interstitial fluid and thereby clearing various neurotoxic metabolites, including ROS and pro-inflammatory cytokines. More specifically, chronic unpredictable mild stress (CUMS) causes a reduction in the polarization of aquaporin 4 (AQP4) expression at the astrocytic level, thus resulting in a downstream functional inhibition of the glymphatic system. This then leads to ROS accumulation and subsequent microglial activation and thus NOD-, LRR-, and pyrin domain-containing protein 3 (NLRP3) inflammasome overexpression and downstream release of pro-inflammatory cytokines, including TNF-α, IL-1α, and prostaglandin E2 (PGE2), which in turn impact the glymphatic system. This neuroinflammatory pathway, along with the classical pathway of defective monoaminergic neurotransmission (regulated by AQP4 and impacted by the established pro-inflammatory habitus), represents the pathogenetic pivot of major depressive disorder (MDD) [42]. The impact of these molecular mechanisms is confirmed in the significant antidepressant efficacy of nonsteroidal anti-inflammatory drugs (NSAIDs) and of biological anti-inflammatory drugs (TNF or cytokine inhibitors), even in non-dermatologic patients [40,41].

### 1.5. Tight Junctions Frailty: A Gateway to Skin and Cerebral Inflammation?

Crucial for the establishment of a skin–brain etiopathogenetic loop is the sleep–wake cycle-disruption-related reduced expression of tight junction (TJ) proteins that make up the TJ complex. In fact, the TJ complex is essential to maintaining cerebral endothelial structural and functional integrity and thus selective BBB permeability. Although the resulting barrier frailty is induced by a chronic reduction in hours of sleep, it can be reversed through the restoration of an optimal sleep–wake cycle [43]. Moreover, TJ proteins are also highly represented in the epithelial barriers of the mucosa and skin, where they regulate the flow of water and solutes between keratinocytes. They also shield the skin from allergens and microbes’ penetration and regulate keratinocyte proliferation. The hypo-expression of these proteins is thus associated with the onset of skin diseases, most notably atopic dermatitis (AD) and psoriasis [44]. The chronic sleep deprivation typical of patients suffering from these pathologies due to junctional deconstruction appears to promote the pathophysiological synergy in the interchanging of ROS and pro-inflammatory cytokines between the skin and the brain. This helps to explain the two-way communication between the skin and mental disorders.

### 1.6. Pathophysiological OS–Inflammation Synergy as a Prerequisite for Etiopathogenetic Synergy between Mental and Organic Disorders

The evidence described in the previous subsections along with the pathophysiological correlation between excessive inflammation as well as structural and functional brain changes pathogenetic to humoral symptomatology then prompted the need to identify a true inflammatory phenotype in patients with mood disorders, which includes central obesity risk, migraines, rheumatoid arthritis, hypothyroidism, adult-onset diabetes, inflammatory skin, and intestinal diseases alongside lifestyle factors [45]. Along with inflammation, OS is a common pathophysiological denominator between inflammatory skin diseases, melanogenesis disorders, skin aging, and chronic psychological stress or neuropsychiatric disorders. It is likely sustained by the concurrent involvement of the renin-angiotensin system along with the autonomic nervous system as well as the HPA axis [46,47]. OS also seems to play a core role in the pathogenesis of psychiatric disorders, particularly in the presence of stressful stimuli in the early stages of life [38,41,42,48,49]. Among mental illnesses, OS in particular plays a crucial role in the pathogenesis of depression because of increased hippocampal sensitivity to oxidative damage. Evidence for this is the finding in depressed patients of malondialdehyde (MDA), nitric oxide (NO), and protein thiol groups (PTGs) overexpression, alongside hypo-expression of total antioxidant status (TAS) [41]. There is a growing pathophysiological interest in neuroendocrine–immune interactions in PTSD. It is a debilitating syndrome triggered by having experienced a terrifying or stressful life experience, which is recurrently relived in the negative feelings associated with the traumatic event [50]. This interest has led to the etiopathogenetic elucidation of comorbid chronic somatic conditions on an inflammatory or autoimmune basis [51] and is particularly relevant in the conflict between Russia and Ukraine.

**Figure 1:**
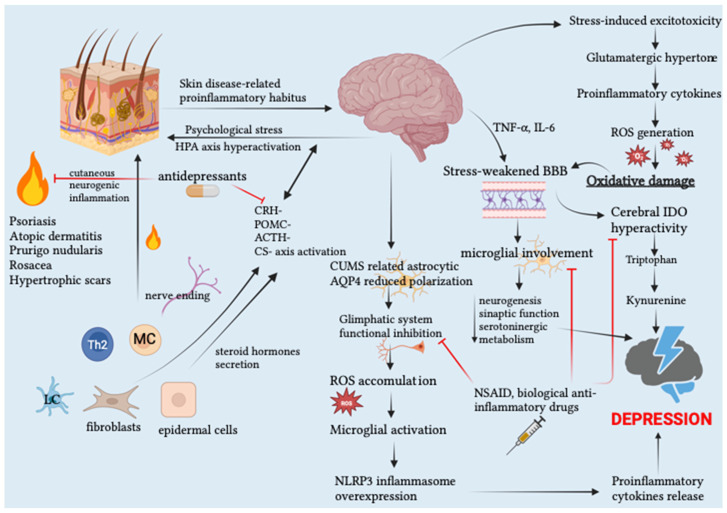
Schematic representation of the complex network of pathophysiological pathways governing the etiopathogenetic bidirectionality between inflammatory skin diseases and mental disorders. More specifically, on the one hand, the phenomenon of stress-induced cutaneous neuroinflammation is emphasized in the context of inflamed skin, which can be considered for all intents and purposes as a neuro-immuno-endocrine organ. On the other hand, the triggering role of skin-derived pro-inflammatory cytokines in initiating the kynurenine pathway and microglial activation in the brain is depicted. These events, together with stress-related glymphatic system dysfunction and thus further pro-inflammatory stimulation, support the inflammatory theory of depression. Significant and corroborating the above physiopathological mechanisms is also the illustration of the cutaneous anti-inflammatory action by antidepressants and, as the downside, the anti-depressant action of NSAIDs and anti-inflammatory monoclonal antibodies. Legend: ACTH, adrenocorticotropic hormone AQP4, Aquaporin-4; BBB, blood–brain barrier; CRH, corticotropin releasing hormone; CUMS, chronic unpredictable mild stress; HPA, hypothalamic–pituitary–adrenal axis; IDO, indolamine 2,3-dioxygenase; IL, interleukin; LC: Langerhans cells; MC, mast cells; NSAID, Non-steroidal anti-inflammatory drugs; POMC, proopiomelanocortin; ROS, reactive oxygen species; TNF, tumor necrosis factor. Created with BioRender.com.

Assuming the aforementioned leading role of OS and its related molecular mechanisms mainly involved in such pathophysiological interplay, our review primarily aims to shed light, from a clinical outlook, on the mutual etiopathogenetic influence between psychological stress/other psychiatric disorders and skin conditions of various matrix, thus supplementing this discussion with a special focus on the OS impact in PTSD in the context of its etiopathogenetic involvement regarding the onset of various skin conditions and, to close, providing an overview concerning potential OS and inflammation biomarkers useful for early detection or prevention of stress-related disorders. Figure 1 represents the complex network of pathophysiological pathways governing the etiopathogenetic bidirectionality between inflammatory skin diseases and mental disorders.

## 2. Discussion

Skin disorders and psychological stress/other neuropsychiatric disorders.

### 2.1. Autoimmune Skin Conditions

#### 2.1.1. Psoriasis

Among chronic inflammatory skin diseases, to date, psoriasis is the most widely investigated regarding the mutual etiopathogenetic influence on mental illnesses. Interest in this regard has been steadily increasing especially in the last decade. Skoie et al. laid the groundwork regarding the evaluation of the interplaying etiopathogenetic link between psoriasis and chronic fatigue, which in turn is influenced by other mental health disorders, such as depression and sleep disorders [52]. They identified fatigue as a prominent feature of the so-called biological model of “sickness behavior”, whose associated pro-inflammatory innate immune response (mediated by monocytes–macrophages and dendritic cells) indicates IL-1β as playing a key role in promoting sickness-inducing effects subject to specific receptor interaction (Interleukin 1 receptor, type I; IL1RI) in the brain. The biological agents used in psoriatic patients also show a promising effect on fatigue. Furthermore, as part of the innate immune response, OS appears to play a key pathophysiological role to be investigated by bridging psoriasis and fatigue [52].

**Figure 2:**
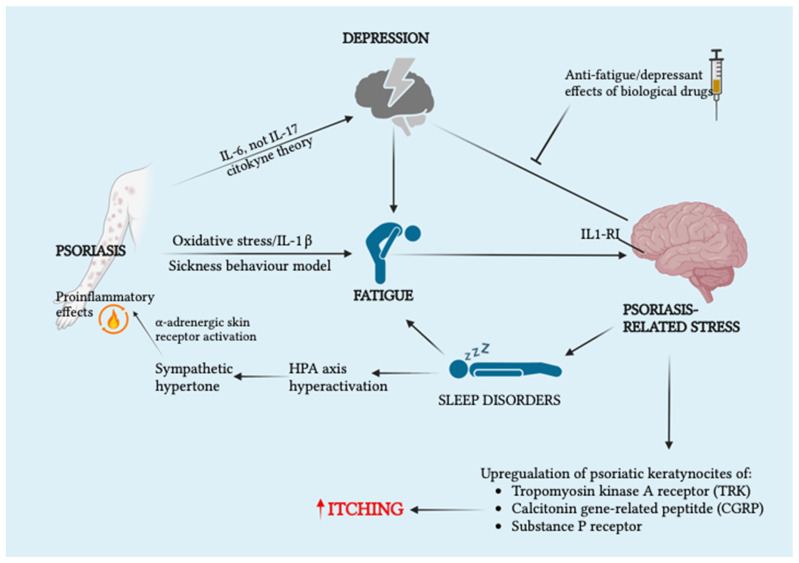
Illustration of the network of pathophysiological mechanisms supporting the etiopathogenetic bidirectionality of psoriasis–neuropsychiatric disorders within the recently recognized skin–brain psoriatic axis. Additionally, relevant here is the antidepressant and anti-fatigue pharmacological effect of biological anti-inflammatory drugs used in psoriatic patients. Legend: HPA, hypothalamic–pituitary–adrenal axis; IL, interleukin. Created with BioRender.com.

Regarding the mutual etiopathogenetic association between psoriasis and its psychological and mental comorbidities [53,54] (above all depression, anxiety, and suicidal ideation and behavior), the main overlapping biological (or more fittingly labeled psychodermatological) mechanism has been identified in systemic inflammation and subsequent pro-inflammatory habitus in the central nervous system (with leading involvement of IL-6) configuring the already-discussed “cytokine theory of depression” [54]. This theory calls into play various pathways such as melatonin dysregulation and related inflammatory cytokine upregulation, kynurenine metabolism, HPA axis hyperactivation, and consequent neurotransmitter dysfunction, associated with worsening neuronal plasticity and reduced hippocampal volume [54]. Surprisingly, the cytokine theory apparently does not involve IL-17, another pivotal cytokine in psoriatic skin inflammation [55]. From a clinical and therapeutical perspective, Fleming et al. reviewed the statistically significant antidepressant effect of selected biologics used in the treatment of moderate to severe psoriasis [56]. Regarding more properly psychological comorbidity, it has been reported that sympathetic nervous system stress-related activation and subsequent alpha-adrenergic receptor activation appear to have a pro-inflammatory effect at the skin level, counterbalanced by the upregulation of the regulatory cytokine IL-10, which is beta-2 adrenergic receptor activation-mediated [54]. Furthermore, exacerbation of pruritus in psoriatic patients has been etiopathogenetically related to stress-induced upregulated expression of certain receptors (tropomyosin receptor kinase A, calcitonin gene-related peptide receptor, and substance P receptor) in keratinocytes of psoriatic plaques [57]. This systemic inflammatory substrate results in the onset of longer-range neuropsychiatric comorbidities and beyond, thus justifying the more recent recognition of psoriasis as a true systemic disease [58,59]. In this context, the etiopathogenetic bidirectionality between psoriasis and sleep disorders has been investigated. In more detail, the key pathophysiological event is HPA axis hyperactivation, which is induced by stress related to psoriatic symptomatology and maintained by sleeplessness symptoms. The downstream effects of this conserved hyperactivation and thus sleep loss will involve pro-inflammatory cytokine hyper-release and sympathetic hyperactivity [59]. The interrelated pathophysiological aspects outlined so far have been recognized as playing a key role in the skin–brain axis in psoriatic patients [60]. This has led to a renewed interest in the multidisciplinary approach of this condition, actively involving psychiatrists, dermatologists, and primary care physicians in adhering to recommendations for clinical practice so as to improve the quality of life of these patients and also reducing related health care spending [61]. Figure 2 represents the network of pathophysiological mechanisms supporting the etiopathogenetic bidirectionality of psoriasis–neuropsychiatric disorders.

#### 2.1.2. Alopecia Areata

A similar etiopathogenetic bidirectionality with mental disorders is also found for alopecia areata (AA). In their review, Kuty-Pachecka highlighted a traumatic situation involving severe mental stress as a prodromal event for the onset and clinical course of AA. The social burden of such a long-lasting, even more extensive skin disorder leads to a range of psychiatric illnesses, such as depressive disorder (most commonly found in dermatological patients), followed by personality disorders and generalized anxiety disorder. Interestingly, a lower frequency in the onset of schizophrenia was shown in patients suffering from AA [62]. A subsequent review revealed the etiopathogenetic role of pre-onset psychological stress in AA, highlighting the acute emotional stress involved in this autoimmune disorder. In such view, the biological mechanisms mainly involved are peripheral HPA axis hyperactivation and acute-stress-induced cholinergic hypertone, leading hair follicle immune cells to experience proinflammatory cytokines hyper-release and resultant disease onset in genetically predisposed individuals [63]. At the follicular level, psychological stress would promote entrance into the catagen phase mainly through two mechanisms: (1) mast cell degranulation induced by the hyperactivated peripheral HPA axis and (2) neurogenic inflammation induced by overexpression of substance P in dermal nerve fibers. Consequently, an increasingly detailed understanding of these pathophysiological aspects is leading to the recognition of the importance of psychotherapeutic interventions in such patients [64].

#### 2.1.3. Immunobullous Skin Diseases

Regarding bullous pemphigoid (BP), an etiopathogenetic bidirectionality is reported in the literature, mainly implicating various neurological disorders (multiple sclerosis, neurodegenerative diseases, cerebrovascular disorders, epilepsies, infectious meningitis, and encephalitis). The two BP autoantigens (BP180 and BP230) play a key role in triggering a cross-reactive autoantibody immune response between the skin and the nervous system. This results in the onset of BP and neuroinflammation or neurodegeneration, as evidenced by the finding of circulating IgG autoantibodies in neurological patients. Although much weaker, an etiopathogenetic association of BP has also been found with various psychiatric disorders (including schizophrenia, uni- and bipolar disorders, personality disorders, depression, and psychosis), leading also in that case to the hypothesis of a neuro–cutaneous cross-reactive immune mechanism [65]. There is also an etiopathogenetic bidirectionality between pemphigus and mental disorders (above all, anxiety and depression), with an associated presumed direct influence of the latter on the clinical severity of skin diseases. Alongside psychiatric comorbidities related to the disease burden, depression may be a side effect due to the chronic use of systemic corticosteroids as first-line therapy in these patients. On the other hand, the supposed bidirectionality seems to be justified by the postulated proinflammatory trigger role of pre-onset psychological stress related to preceding adverse life events [66].

#### 2.1.4. Vitiligo

The first-ever evidence of a specific psycho–dermatologic connection in vitiligo showed a direct correlation between this depigmenting skin disorder and depression, with depression increasing in clinical severity in proportion to the time elapsed since disease onset and the representativeness of visible lesions. On the other hand, in the context of the etiopathogenic vitiligo–psychological stress bi-directionality, the widely accepted biological mechanism identifies stress as playing a triggering role in the HPA axis activation and subsequent inflammatory response. It can also cause melanocytic oxidative damage due to hydrogen peroxide accumulation in the epidermal layer, as also revealed by the high levels of cortisol and low levels of dehydroepiandrosterone (DHEAS), an antioxidant-acting hormone, among vitiligo patients [67].

### 2.2. Chronic Inflammatory Dermatoses

#### 2.2.1. Lichen Planus

Regarding oral lichen planus (OLP), Li et al. pointed out significantly higher levels of anxiety, depression, and stress in OLP patients than in controls, probably triggered by apprehension about the malignant potential of this disorder. However, such mental disorders were recognized as playing a pivotal role in the onset and development of OLP. The putative biological mechanisms involved in this etiopathogenetic bidirectionality invoke OLP-related oral and gut microbiota imbalances and subsequent attempted microbiota neuroimmune regulation along the brain–gut axis, which then likely promote the onset of psychiatric comorbidities. On the other side, given the oral mucosal sensitivity to emotional stimulation, OLP onset could be explained by innate and adaptive immune response dysregulation (especially concerning T helper 1-T-helper 2 shift), which is induced by acute and chronic psychological stress, and OLP immune dysregulation is in turn exacerbated by psychiatric comorbidities [68].

#### 2.2.2. Hidradenitis Suppurativa

In the more recent consideration of hidradenitis suppurativa (HS) as a systemic inflammatory disease, the presumed sharing of inflammatory pathways would represent a biological mechanism useful to explain the etiopathogenetic link between this skin condition and various other comorbidities, including autoimmune and autoinflammatory diseases, metabolic disorders, skin cancer, and psychiatric disturbances [69,70,71,72]. Misitzis et al. showed that psychiatric disorders are by far the most common group of comorbidities in HS patients, and thus, they merit epidemiological and etiopathogenetic investigations [73]. The most notable associations appear to be with major depression, anxiety, bipolar disorder, psychosis, substance abuse disorder, and suicide. These psychiatric comorbidities in HS patients, aside from the psychosocial impact, appear to be the result of biologically shared inflammatory mechanisms that involve the same proinflammatory cytokines [73]. These epidemiological and etiopathogenetic findings involving HS and psychiatric disorders have been further confirmed in a recent review of the literature on this issue [74].

#### 2.2.3. Facial Dermatoses

The vicious etiopathogenetic cycle of facial dermatoses–emotional factors has been frequently investigated in the last decade. Early reviews of the literature confirmed that acne vulgaris is as a psychosomatic and somatopsychic disorder, with psychological stress and anxiety being the main triggering and/or exacerbating event [75]. The biological mechanism hypothetically involved recognizes the peripheral HPA axis as playing a crucial role due to expression at the sebocyte level of functional receptors for various neuroendocrine mediators of the stress response, resulting in upregulation of lipogenesis, androgen metabolism, and pro-inflammatory cytokines. Acne excoriata primarily recognizes a crucial etiopathogenetic role in an underlying psychiatric disorder, including anxiety, depression, obsessive-compulsive disorder, and personality disorder. Regarding rosacea, psychological stress likely induces OS and inflammation with the recruitment of specific leukocyte subsets. The presumed etiopathogenetic bidirectionality between seborrheic dermatitis and emotional stress has rarely been investigated biologically. With regard to herpes simplex virus (HSV) infections, psychological stress is a major trigger for symptomatic recurrence, especially for oral herpes. The biological mechanisms presumably involved call into play stress-induced activation of both the HPA axis and the sympathetic system and consequent over-release of stress hormones (cortisol and catecholamines). This leads to a cytokine shift from Th1 to Th2 with subsequent suppression of Th1 cytokines, which are essential in ensuring the functionality of HSV-specific CD8+ memory T cells, resulting in viral reactivation [75]. On this issue, more recent literature revisions have mainly focused on acne and rosacea. Haber et al. were the first to highlight the systemic impact of rosacea as a skin disorder associated with cardiovascular, gastrointestinal, metabolic, oncological, neurological, and psychiatric comorbidities [76]. Concerning psychiatric comorbidities, a higher incidence of depression and social anxiety was noted in such patients. Social anxiety, along with psychological stress, appears to trigger and exacerbate the disease. This psychosomatic association seems to be due to the priming of similar inflammatory pathways, with the presumed involvement of matrix metalloproteinases (MMPs), as detected by their higher serum levels found in such patients [76]. The vicious etiopathogenetic cycle between rosacea and psychiatric comorbidities was reevaluated by Woo et al., who better elucidated the biological mechanisms underlying the shared inflammatory pathways. These pathways are crucial for such bidirectionality and result in the dysregulation of the gut–brain–skin axis, also manifesting in the alteration of both skin and gut microbiota. Various psychological stressors via neurotransmitter pathways would induce neuropeptide release by enteroendocrine cells, resulting in increased intestinal permeability and thus local and then systemic inflammation. A presumed key role in triggering and sustaining these phlogistic events would be ascribed to IL-17, which is involved in the onset and aggravation of both psychiatric disorders and rosacea [77]. Acne has only recently been investigated from a psychodermatological perspective. Again, there seems to be a bidirectional relationship with psychological stress as well as an association with various psychiatric comorbidities, such as anxiety, fear, social phobia, type D personality, sleep disorders, depression, sexual dysfunction, and suicidal ideation/attempt. Biologically, underlying this etiopathogenetic relationship is the stress-induced release of neuropeptides inducing the production of proinflammatory cytokines and thus inflammation as well as the stress-induced dysfunction of skin barrier, impaired wound healing, and enhanced susceptibility to certain bacterial infections [78]. Psychodermatological interest in acne has also focused on studying specific populations with a high prevalence for this condition because of the high levels of stress to which such populations are exposed as well as assessing the related psychosocial impact of this skin disease. For example, among medical students, stress has been reported to be linked to both the incidence and severity of acne. On the other hand, its psychosocial impact is embodied in the occurrence of anxiety, depression, embarrassment, negative self-image, and low self-esteem. Biologically, the aforementioned mechanisms are also confirmed [79].

#### 2.2.4. Other Herpesvirus-Related Skin Manifestations

Other herpesviruses have also been investigated from the point of view of the etiopathogenetic correlation between viral reactivations and psychological stress. In this regard, a systematic review and meta-analysis performed by Marra et al. shed light on a statistically significant association of varicella-zoster virus (VZV) infection with physical trauma rather than psychological stress among the various nonmedical factors evaluated [80]. In contrast, regarding Epstein–Barr virus (EBV) reactivation, assuming its albeit uncommon dermatological involvement, the etiopathogenetic influence of various psychological stressors has recently been emphasized through evidence of higher EBV antibody titers and increased EBV-DNA shedding in certain populations exposed to high levels of stress, especially chronic stress. This thus confirms that chronic or long-term stress has immunosuppressive action as opposed to the acute or short-term stress-related immunoenhancing role [81,82,83]. Interestingly, from a biological viewpoint, this etiopathogenetic correlation seems to be influenced by the opposing action of sex hormones on the HPA axis, which is downregulated by testosterone and upregulated by estrogen and progesterone [82].

#### 2.2.5. Atopic Dermatitis

AD is a systemic disease, with various non-allergic and neuropsychiatric comorbidities, including anxiety, depression, attention deficit hyperactivity disorder, emotional problems, conduct disorder, and suicidal ideation. Their likelihood is directly proportional to the clinical severity of AD. Once again, there is an etiopathogenetic bidirectionality of these conditions. The biological mechanisms that would plausibly explain these comorbidities involve Th2-mediated systemic inflammatory response along with sympathetic hypertone and HPA dysregulation as a consequence of chronic early-onset inflammation or chronic use of systemic corticosteroids [84]. This etiopathogenetic bidirectionality is further strengthened by the recently revised “moi-peau concept” (or otherwise called the “Ego-Skin concept”), which emphasizes how psyche and skin mutually reflect each other’s needs [57]. The clinical exemplification of what has just been described finds literary resonance in the interplay between itch (a pivotal symptom of several chronic skin disorders, including AD) and psychological stress. This interplay, from a biological point of view, is due to an aberrant parasympathetic response, whereas in AD, this is due to the synergy between HPA axis and neuro-endocrine-immunocutaneous system (NEICS) [57]. The most recent literature advancement in this regard has led to the development of a mechanistic model to explain the alteration of both brain neural networks and the HPA axis in inducing the dysfunctional scratching behavior in AD, which is ultimately responsible for disease progression. More specifically, at an early stage of the disease, the synergistic noradrenergic and HPA axis hyperactivation induced by scratching behavior drives the dysregulation of specific neural circuits. This results in itch cognitive distortion and a reinforcement of scratching behavior, thereby worsening skin lesions. These neural dysfunctions are maintained in late-stage AD, with only the functional change occurring in the HPA axis: upregulated in early stage and presumably directing Th2-skewed immune response and downregulated in late stage and presumably promoting co-dominant Th1 inflammatory shift [85]. In the wake of what has just been reported, further confirmation has come in support of the role of psychological stress in the onset or exacerbation of AD, which is supported by new biological mechanisms that recognize the mast cell as the “neuro-immunoendocrine master player”. Restraint stress appears to activate HPA axis and CRH release, which in turn stimulates brain mast cells to release vascular endothelial growth factor and pro-inflammatory mediators (especially cytokines such as IL-5, IL-6, IL-31, IL-33, and TNF). These mediators are responsible for increased microvascular permeability at the level of the blood–brain barrier and thus the release of these mediators in the circulation. In addition, mast cell activation is further enhanced by the synergistic action of other neuropeptides (such as neurotensin and substance P), which are responsible for CRHR1 upregulation on the human brain mast cell at the hypothalamic and meningeal levels. The same stress-related activation of the peripheral cutaneous HPA axis and subsequent CRH–CRHR1 interaction also expressed on skin mast cells further boosts the brain-primed inflammatory mechanism [86]. Table 1 represents the pathophysiological mechanisms governing the etiopathogenetic bidirectionality in mental–cutaneous disorders, whether proven or supposed.

### 2.3. PTSD: A Systemic Inflammatory Disorder

The etiopathogenetic influence of PTSD on various skin disorders is not a novelty of recent literature. Gupta et al. highlighted the cutaneous manifestations secondary to PTSD, including (1) specific cutaneous sensory perceptions typically representative of the original traumatic event; (2) prominent flushing, profuse night sweating, and pruritus as major skin manifestations related to autonomic hyperarousal and elevated skin conductance in response to neutral stimuli; and (3) dissociative reactions manifesting as self-induced dermatoses such as trichotillomania, acne excoriata, and dermatitis artefacta or as conversion symptoms such as loss of touch and pain or psychogenic purpura [87,88]. The biological mechanisms governing PTSD have been investigated in order to explain the various organ system conditions related to it. At the core of such discussions is undoubtedly the induction of inflammatory pathways by chronic stress and thus sympathetic overactivation [89]. Inflammation and OS are the key pathophisiologic mechanisms underlying PTSD, which is seen as a true systemic or psychosomatic disease due to its close association with various comorbidities (neuropsychiatric, cardiovascular, gastrointestinal, endocrine, metabolic, autoimmune, hematologic, respiratory, muscular, and dermatologic). Inflammation and OS arise simultaneously and influence each other. Evidence suggests an epigenetic (mRNA-mediated) stimulation of PTSD-related neuroinflammation with an associated downregulation of regulatory T cells. The first systemic implication of these biological mechanisms is the increase in the serum levels of various proinflammatory cytokines such as IL-6, interferon-gamma (IFN-γ), IL-1β, IL-17, IL-2, and TNF-α together with increased serum C-reactive protein (CRP). OS-induced (brain-driven) release of adrenal cortisol downregulates inflammation, albeit in the short term. However, it also creates a homeostatic imbalance in digestive microflora, resulting in an upsurge of commensal pathobionts such as *Helicobacter pylori*, the latter being capable of amplifying local and systemic inflammation. In addition, according to “The glucocorticoid-hippocampal atrophy model”, HPA axis long-term stress-related overstimulation would be responsible for neurotoxicity and thus neurodegeneration of certain brain structures, primarily the hippocampus due to its abundance in glucocorticoid receptors, which, by entailing higher concentration of ROS, lead to oxidative damage at such level [90,91]. A further essential pathophysiological mechanism in the origin and development of PTSD that has only recently been better-defined calls in play the crucial role of mitochondrial stress response. Specifically, two stress-induced mitochondrial pathways are recognized. Firstly, HPA axis hyperactivation and the consequent release of glucocorticoids regulates mitochondrial transcription, orienting it towards the stress response through a series of biological mechanisms such as mitochondrial unfolded protein response (UPRmt), mitophagy, apoptosis, alterations in the electron transport chain (respiratory chain), and consequent ROS generation. Secondly, via N-methyl-D-aspartate (NMDA) receptors acting as ion channels, stress-induced glutamatergic hypertone enables the massive intraneuronal calcium ion (Ca^2+^) influx and consequent mitochondrial ion overload. This leads to caspase activation and on the other hand to the mitochondrial membrane potential collapse, thereby reducing adenosine triphosphate (ATP) generation, which is counterbalanced by ROS hypergeneration. The final biological result is cell death (by apoptosis and oxidative damage) and consequent ROS explosion in the brain, with considerable organ damage due to its reduced antioxidant defenses combined with the richness of lipid structures as well as the high demand for oxygen and energy [92].

### 2.4. Inflammatory and Oxidative Markers: Wide-Ranging Clinical Potential

Circulating inflammation and OS markers may play an important role in neuropsychiatric disorders and somatic affections (including dermatological ones) for diagnostic, prognostic, and follow-up purposes. There are individual differences in behavioral patterns of stress response, known as “coping styles”, especially from a physiological and biochemical perspective. These individual differences in coping strategies translate into a different, individualized clinical–therapeutic impact of the negative consequences of stress, such as mental and peripheral disorders. As a result of the aforementioned, the identification of coping-style markers, relating above all to individual differences in stress-related inflammatory and oxidative mechanisms, is key to identifying an individual phenotype of stress sensitivity/resistance and thus to accurately estimating the risk of incurring in stress-related disorders [93]. Furthermore, measuring ROS in biological fluids is particularly difficult due to their short half-life compared to inflammatory markers. Research in this area has thus focused on sub-products, namely products resulting from the interaction between ROS and cellular molecular structures. It has also focused on identifying markers reflecting the cellular antioxidant defense response, such as the nuclear transcription factor erythroid-2 (Nrf2), which is the main transcriptional regulator of cell homeostasis through its interaction with the promoter sequence of cytoprotective genes, known as the antioxidant response element (ARE) [94]. Regarding OS markers, a promising role, albeit non-specific and yet to be investigated in depth, would be assigned to advanced oxidation protein products (AOPPs), which are characteristically accumulated in most inflammatory and immune-mediated diseases, including dermatological ones such as psoriasis, vitiligo, melanoma, and oral cavity cancer [95]. Specifically concerning PTSD, the most recent systematic review and meta-analysis on inflammatory and OS markers in this disorder revealed significantly higher serum levels of CRP, IL-6, and TNF-α in PTSD patients compared to healthy controls. Conversely, no OS markers were found to be associated with PTSD [96].

## 3. Conclusions

Skin disorders and mental illnesses represent two pathological spheres of considerable clinical–epidemiological and socioeconomic importance. New diagnostic–therapeutic approaches are needed that aim at “killing two birds with one stone”. This is the case of new therapeutic frontiers aiming at employing antidepressant and anti-inflammatory drugs, both conventional and biological, as the only presides capable of treating the disorders of these two interconnected comorbidities. Future medical research needs to clarify the pathophysiological mechanisms biologically involved in this two-way etiopathogenetic communication. Additionally, epigenetic research on potential therapeutic targets should investigate the aberrant epigenomic mechanisms responsible for molecular transduction from the psychic to the biological world, ultimately aiming toward precision medicine within PNEI. In this context, given the pleiotropic effects of melatonin, a promising new therapeutic solution from unconventional medicine could be the synthesis of a melatonin form with high transmembrane MT1 and MT2 receptor affinity. This would help to upregulate the anti-inflammatory/antioxidant action while disfavoring the pro-inflammatory IL-17 cytokine family-mediated one, which is crucial in both autoimmune and neurodegenerative diseases. Similarly, on the diagnostic–prognostic front, the stress-response behavioral patterns need to be defined along with specific markers of coping styles (both inflammatory and oxidative). The goal would be to provide a detailed framework of the individual stress-resistance/sensitivity phenotype. This would then pave the way for a new frontier of personalized medicine in the psychodermatological field.

## Figures and Tables

**Table 1:** The pathophysiological mechanisms governing the etiopathogenetic bidirectionality in mental-cutaneous disorders, whether proven or supposed.

Skin Disease	Psychiatric Comorbidities	Pathophysiological Mechanisms
Alopecia areata	Depressive, personality, and generalized anxiety disorders	(1) Acute stress-related HPA axis hyperactivation, subsequent mast cell degranulation, and catagen phase promotion; (2) Acute stress-related substance P overexpression and neurogenic inflammation promoting catagen phase entry; (3) Acute stress-related cholinergic hypertone and subsequent hyper-release of inflammatory cytokines by immune cells in the hair follicle.
Bullous pemphigoid	Schizophrenia, uni and bipolar disorders, personality disorders, depression, and psychosis	Cross-reactive neurocutaneous autoimmune (BP 180/BP230-mediated) response and subsequent neuroinflammation-neurodegeneration.
Pemphigus	Anxiety and depression	Proinflammatory habitus triggered by psychological stress related to previous adverse events.
Vitiligo	Depression	Stress-related HPA axis hyperactivation -> inflammation -> melanocytic oxidative damage.
Oral lichen planus	Anxiety, depression, and stress	(1) OLP-related oral and gut dysbiosis -> attempted microbiota neuroimmune regulation -> psychiatric disorders; (2) Stress-induced innate and adaptive immune dysregulation (Th1-Th2 shift) -> OLP.
Hidradenitis suppurativa	Major depression, anxiety, bipolar disorder, psychosis, substance abuse disorder, and suicide	Sharing of inflammatory pathways and involvement of the same cytokines.
Acne	Stress, anxiety, depression, obsessive-compulsive disorder, personality disorder, sexual dysfunction, and suicidal ideation/attempt	(1) Stress-related HPA axis activation and stress response at sebocyte level -> upregulation of lipogenesis, androgen metabolism, and pro-inflammatory cytokines; (2) Stress-induced dysfunction of skin barrier, wound healing, and enhanced susceptibility to certain bacterial infections.
Rosacea	Depression and social anxiety	(1) Shared inflammatory pathways with presumed involvement of matrix metalloproteinases (MMPs); (2) Dysregulation of the gut–brain–skin axis, also manifesting in alteration of both skin and gut microbiota.
Herpes simplex virus	//	Emotional stress-induced HPA axis and sympathetic system activation -> stress hormones-induced Th1-Th2 shift -> HSV-specific CD8+ memory T-cells functional inhibition -> viral reactivation.
Epstein–Barr virus	//	Chronic stress-induced HPA axis hyperactivation.
Atopic dermatitis	Anxiety, depression, attention deficit hyperactivity disorder, emotional problems, conduct disorder, and suicidal ideation	- Th2-mediated systemic inflammatory response, sympathetic hypertone, and HPA dysregulation as a consequence of chronic early-onset inflammation or chronic use of systemic corticosteroids; - Stress-induced functional synergy between HPA axis and neuro-endocrine-immunocutaneous system (NEICS) and aberrant parasympathetic response in supporting itch–stress interplay.

## Data Availability

Not applicable.
